# Assessment of p16 and Ki67 Immunohistochemistry Expression in Squamous Intraepithelial Lesion with Cytohistomorphological Correlation

**DOI:** 10.30699/ijp.2020.112421.2208

**Published:** 2020-07-16

**Authors:** Apurv Ghosh, Nirupama M, Nandan Padmanabha, Hema Kini

**Affiliations:** Department of Pathology, Kasturba Medical College, Mangalore, India

**Keywords:** HSIL, LSIL, Squamous intraepithelial lesion

## Abstract

**Background & Objective:**

Cervical cancer is the most common cancer in women worldwide with high mortality, necessitating quicker diagnostic methods. We wish to enhance the existing cervical biopsies of Squamous Intraepithelial Lesions (SIL) using p16 and Ki67 as surrogate markers to assess correlation between its positivity and histological grade of the lesion.

**Methods:**

Analysis of p16 and Ki67 expression was done on 31 histopathologically diagnosed cases of SILs. Positive expression of p16 was assessed based on a scoring system and compared with histology and cytology. Ki67 expression was studied and the correlation was observed with degree of dysplasia. Twenty cases of chronic cervicitis was assigned to the control group for comparison.

**Results:**

Cases of HSIL showed greater expression of p16 as compared to LSIL. Sensitivity of p16 for HSIL was higher than that for LSIL. The specificity for HSIL and LSIL was 100%. Ki67 expression correlated well with the degree and level of dysplasia with a significant P-value of 0.002.

**Conclusion:**

p16 and Ki67 positivity of SILs should point towards further evaluation. The expressions of p16 and Ki67 are useful markers for confirmation of SILs and in predicting HPV infection which can be further confirmed by HPV DNA testing.

## Introduction

Carcinoma cervix is one of the most common cancers among women worldwide and is the second most frequent type of cancer among Indian women (1). In India, an estimated 67,477 deaths have been attributed to cervical cancer (2). Early detection of the precursor lesions is therefore of paramount importance to reduce the mortality burden of the disease (3). Various screening modalities are being used to identify high risk patients and guide follow up and further management (4). The Papanicolaou (pap) cervical cytology test has been routinely used since 1960 to screen precursor lesions of the cervix and the diagnostic criteria have been updated since the introduction of the Bethesda system of reporting cervical cytology (5).

The earlier terminology of cervical intraepithelial neoplasia (CIN) and its categorisation into three groups (CIN 1,2,3) were riddled with interobserver variability (6-8). With the introduction of the two-tiered system to classify precursor lesions as squamous intraepithelial lesion (SIL), high grade or low grade reproducibility improved, but the quest to improve diagnostic accuracy has led to research with various immunohistochemical markers that target the basic mechanism in the pathogenesis of premalignant lesions of the cervix (9,10) The Human papilloma virus (HPV) infection is responsible for SIL and its progression to invasive carcinoma has been well established. The high-risk 'HPV types 16 and 18’ account for the majority of the women being affected (11). In the life cycle of the virus, the expression of oncoproteins E6 and E7 during the “transformation” phase, leads to inhibition of the tumour suppressor proteins *p53* and *Rb* gene. Overproduction of E2F leads to cyclin D1 inhibition and ultimately leading to p16 overexpression in the infected cells (12).

The overexpressed p16 can be detected using immunohistochemistry (IHC) and used in improving diagnostic accuracy (12-14). Another such marker is Ki67, which is expressed during active phases of the cell cycle indicating cellular proliferation (12,15). Over the years, a number of markers have been studied alone or in combination to best analyse, precursor lesions of the cervix to improve diagnostic accuracy (9,16,17). 

In resource poor centres which cater to an economically challenged population, the feasibility of running an entire battery of markers may not be possible (10,18). In this study immunohistochemical expression of p16 and Ki67 were analysed in histologically and cytologically diagnosed cases of squamous intraepithelial lesions, and to correlate its association with high and low-grade lesions.

##  Materials and Methods

**Case Selection**

A retrospective study was conducted between January 2015 and April 2017. Thirty-one cases were selected with histopathological diagnosis of cervical intraepithelial lesion (CIN 1,2,3) or squamous intraepithelial lesion (HSIL or LSIL). The specimens included hysterectomy specimens, conisation or punch biopsy samples. Tissue blocks with inadequate material, excessive haemorrhage or necrosis were excluded. The Pap smears were obtained for the cases wherever possible. Histopathological examination was performed at the Department of Pathology, at a tertiary centre in coastal region of India. All the H&E slides were reviewed and a diagnosis of either high grade squamous intraepithelial lesion or low grade squamous intraepithelial lesion was assigned, based on the WHO criteria. The corresponding Pap cervical cytology smears were also reviewed wherever possible and the diagnosis was given, according to the Bethesda System of reporting cervical cytology. Twenty cases of histopathologically diagnosed cases of chronic cervicitis were taken as control for p16 and Ki67 immunostaining.

**Immunohistochemistry**

The representative H&E section was selected for immunohistochemistry with p16 and Ki67. IHC was performed on formalin-fixed, paraffin-embedded tissue. Counterstaining was done using Meyer’s hematoxylin.

**Interpretation**

Positivity for p16 was considered when there was block staining of nuclear, along with or without cytoplasmic staining (19). The degree of intensity of the stain, parabasal involvement, pattern of staining (focal or diffuse), and percentage of positive staining dysplastic cells were analyzed. Each parameter was graded, and a combined score was used to determine positive or negative result using the criteria used by Alshenawy H. *et al.* (12,20) (Table 1). For p16 expression to be considered positive a total combined score of >3 was required.

Ki67 proliferation index is defined as the percentage of Ki67 positive cells. Grade 1+, 2+, and 3+ were given when the Ki67 index was below 5%, 5–30%, and greater than 30%, respectively by observing nuclei of 200 epithelial cells located across the whole epithelial layer in high-power field as used by Alshenawy H. *et al.* (12).

**Table 1:** Immunohistochemistry scoring for p16

p16 immunostaining grade:		Score
**Percentage of positive cells (%)**	<5%	0
	5-49%	1
	50-80%	2
	>80%	3
**Intensity of reaction**	No reaction	0
	Weak	1
	Variable	2
	Strong	3
**Cellular reaction pattern**	No reaction	0
	Focal	1
	Diffuse	2
**Total**		
**Negative (0–3)** **Positive (4–8)**		

**Statistical Analysis**

Data analysis was done using SPSS 17 (SPSS Inc., Chicago, Ill. USA); collected data was analysed by both descriptive and inferential methods. Descriptive method such as frequency and percentage were calculated to summarise the data. Sensitivity, specificity, positive predictive value and negative predictive value along with agreement were calculated. 

## Results

**Fig 1:**
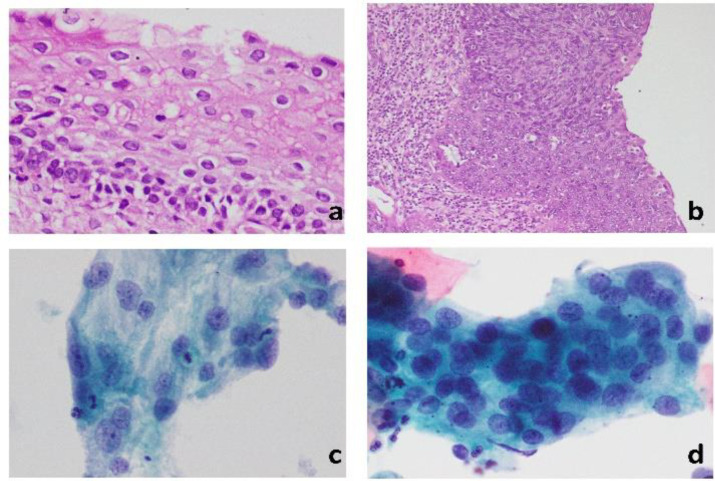
a: Low grade squamous intra-epithelial lesion (LSIL) H&E 200X

**Fig 2:**
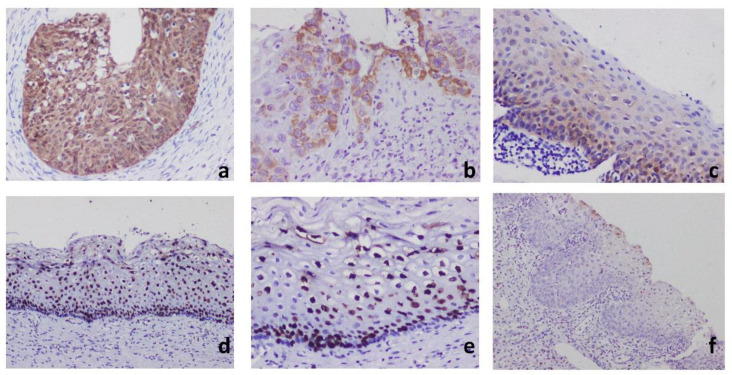
a:16 positive (score 8) with diffuse strong expression in HSIL

The age of the patients ranged between 28 and 68 years with majority of the patients being below 50 years of age. Majority of the cases of SIL were with parity of 2 or more. Most common clinical presentation was bleeding per vaginum. Of the total 31 cases, 12 were diagnosed as HSIL while 19 were diagnosed as LSIL on histopathology (Figure 1). Corresponding Pap smear was unavailable for 5 cases. The overall absolute correlation between cytology and histopathology was 58.1%. HSIL was under-reported with 6 cases being reported out of 12 histopathologically diagnosed cases. Five ASC-H and one ASCUS assigned cases on cytology were upgraded to HSIL on histopathology. LSIL cases correlated well, with 13 out of 19 cases being assigned on cytology while one ASCUS case being upgraded to LSIL on histology. According to the criteria used by Alshenawy H *et al.*, in the present study, 58.3% (7/12) cases of HSIL showed absolute p16 positivity while 26.3% (5/19) cases of LSIL showed p16 positivity (Figure 2).

b: High grade squamous intra-epithelial lesion (HSIL) H&E 100X c: LSIL pap 400X d: HSIL pap 400X

b: p16 positive (score 4) with focal variable expression in HSIL c: p16 positive (score 4) with focal positive expression in LSIL

d: Ki-67 grade 2+ in HSIL e: Ki-67 grade 2+ with full thickness expression in HSIL f: Ki-67 negativity in LSIL

In the present study a total of 38.7% (12/31) cases of SILs showed absolute p16 positivity. Sensitivity of p16 was 38.7% with 100% specificity for SILs. Positive predictive value was 100% while negative predictive value was 51.3%. Overall agreement between was 62.74. Of 19 negative cases of p16, 4 cases showed absolute negativity with a score ‘0’. While 15 cases had a score of 2 or more. Among all the SIL cases some amount of p16 activity was observed in 87% of the cases. The expression of Ki67 was “grade 1” in 67.7% of the cases with anatomical expression in the respective cases. The Ki67 expression in the middle and superficial third of the epithelium correlated well with the histopathological diagnosis with a P-value of 0.001 and 0.002 respectively. The control group comprising of diagnosed cases of chronic cervicitis showed no expression with p16 and with 5 out of 20 cases showing <5% expression of Ki67 (Tables 2 and 3).

**Table 2:** p16 immunostain scoring in LSIL and HSIL

p16 immunostaining		HSIL n (%)	LSIL n (%)	Chronic Cx n (%)
Percentage of positive cells (%)	0(<5%)	5 (41.7%)	11(57.9%)	20 (100%)
	1(5-49%)	4(33.3%)	6(31.6%)	
	2 (50-80%)	3 (25.0%)	2(10.5%)	
Intensity of reaction	0 (No reaction)	1 (8.3%)	3 (15.8%)	20(100%)
	1(Weak)	3 (25.0%)	10 (52.6%)	
	2 (Variable)	4 (33.3%)	4 (21.1%)	
	3 (Strong)	4 (33.3%)	2 (10.5%)	
Cellular reaction pattern	0 (No reaction)	1 (8.3%)	3 (15.8%)	20(100%)
	1 (Focal)	10 (83.3%)	16 (84.2%)	
	2 (Diffuse)	1 (8.3%)	0 (0%)	
p16 Negative (0–3)	0	1 (8.3%)	3 (15.8%)	20 (100%)
	2	3 (25.0%)	7 (36.8%)	
	3	1 (8.3%)	4 (21.1%)	
Positive (4–8)	4	4(33.3%)	2 (10.5%)	
	5	0 (0%)	2 (10.5%)	
	6	2 (16.7%)	1(5.3%)	
	7	1 (8.3%)	0 (0%)	

**Table 3:** Ki-67 grading in HSIL and LSIL

Ki67	HSIL n (%)	LSIL n (%)	Chronic Cx
0	0(0%)	0(0%)	16(80%)
1 (<5%)	6 (50.0%)	15 (78.9%)	4(20%)
2 (5-30%)	4 (33.3%)	4 (21.1%)	0(0%)
3 (>30%)	2 (16.7%)	0 (0%)	0(0%)

**Table 4:** Comparison of p16 and Ki67 expression between various studies and present study

Reference	n	LSIL			HSIL		
		n	p16	Ki67	n	p16	Ki67
**Present study**	51	19	5	19	12	7	12
**Hebbar ** ***et al.*** ** (** 13 **)2017**	50	10	5	7	20	16	19
**Alshenawy A ** ***et al.*** ** (** 8 **) 2014**	75	15	4	6	48	32	48
**Xing Y. ** ***et al.*** ** (** 19 **) 2017**	95	45	11	16	40	35	38
**Kanthiya K. ** ***et al.*** ** (** 16 **) 2016**	243	106	11	24	61	48	46

## Discussion

Although histopathology remains the gold standard for the diagnosis of SILs, immune-histochemistry can be helpful in limited tissue biopsies and eliminating the interobserver variability. Recently p16 has gained popularity not only in typing the lesion but also in predicting treatment response (21). Several authors have studied p16 expression using different positivity criteria in both preinvasive and invasive squamous carcinomas (22–25). In this present study the grading used for p16 positivity, was proposed by Songkhun V. *et al.* and later used by Alshenawy H. *et al.* (12,20).

In the present study the sensitivity for HSIL was 58.3% and for LSIL was 26.3%. While other authors found p16 expression to be between 45% and 100% for HSIL and between 10% and 70% for LSILs (26). In the study by Eleuterio J. *et al*. in 2007, a positive p16 reaction was seen in 92.3% cases and 15.4% cases of HSIL and LSIL respectively (27). Diane M.C. *et al.* observed 80.9% and 19.36% positivity with p16 in HSIL and LSIL respectively (18). The authors also stated a sensitivity of 86.9% and a specificity of 87.7% with p16 for HSIL (28). Srivastava S. *et al.* observed 100% positivity for p16 for both LSIL and HSIL (12). Xing Y. *et al.* observed 24.4% and 87.5% positivity with p16 in LSIL and HSIL respectively (29). In the study by Leite P. *et al.* p16 positivity observed was 12.8% and 72.1% for LSIL and HSIL respectively. They also observed a significant relation between p16 positivity and recurrence with P-value of 0.018 (30). Comparative analysis of p16 and Ki67 with various recent studies and the present study is depicted in Table 4.

The degree of dysplasia correlated with Ki67 expression, in most of the cases had grade 1 expression with 33.3% (4/12) cases of HSIL having grade 2 and 16.7% (2/12) cases of HSIL having grade 3 expression of Ki67. It was also observed that Ki67 expression was extending into the superficial third of the epithelial layer while LSIL demonstrated Ki67 expression which was limited to the basal third. These findings were similar to those by Srivastav S. *et al*. and Hebbar A. *et al.* (22,23).

## Conclusion

All the SILs showed 100% Ki67 expression with a comparatively good expression of p16 in HSIL followed by LSIL. Such cases with positive p16 and high Ki67 expression should be further evaluated for HPV DNA typing as a routine protocol although not performed in the present study.

Sensitivity and specificity of p16 expression was low as compared to other studies. HPV infection status with p16 expression were not available due to resource constraints. Although we have considered block positivity for p16 as true positive, a uniform grading criterion for establishing p16 expression would be effective in interpretation with consensus.
